# The role of DPYD and the effects of DPYD suppressor luteolin combined with 5‐FU in pancreatic cancer

**DOI:** 10.1002/cam4.70124

**Published:** 2024-08-19

**Authors:** Hiroyuki Kato, Motonori Sato, Aya Naiki‐Ito, Shingo Inaguma, Makoto Sano, Masayuki Komura, Yuko Nagayasu, Kuang Xiaochen, Akihisa Kato, Yoichi Matsuo, Hideaki Ijichi, Satoru Takahashi

**Affiliations:** ^1^ Department of Experimental Pathology and Tumor Biology Nagoya City University Graduate School of Medical Sciences and Medical School Nagoya Japan; ^2^ Department of Anesthesiology Nihon University School of Medicine Tokyo Japan; ^3^ Department of Gastroenterology and Metabolism Nagoya City University Graduate School of Medical Sciences and Medical School Nagoya Japan; ^4^ Department of Gastroenterology Surgery Nagoya City University Graduate School of Medical Sciences and Medical School Nagoya Japan; ^5^ Department of Clinical Nutrition Center, Graduate School of Medicine the University of Tokyo Hongo Tokyo Japan

**Keywords:** 5‐fluorouracil, combination drug therapy, dihydropyrimidine dehydrogenase, luteolin, pancreatic cancer

## Abstract

**Background:**

Despite advances in the treatment of cancer, pancreatic ductal adenocarcinoma (PDAC) remains highly lethal due to the lack of effective therapies. Our previous study showed that Luteolin (Lut), a flavonoid, suppressed pancreatocarcinogenesis and reduced the expression of dihydropyrimidine dehydrogenase (DPYD), an enzyme that degrades pyrimidines such as 5‐fluorouracil (5‐FU), in PDACs. In this study, we investigated the role of DPYD and evaluated the therapeutic potential of combining 5‐FU with Lut in PDACs.

**Methods and Results:**

PDAC cells overexpressing DPYD showed increased proliferation, and invasiveness, adding to the resistance to 5‐FU. The xenograft tumors of DPYD‐overexpressing PDAC cells also exhibit enhanced growth and invasion compared to the control xenograft tumors. RNA‐seq analysis of the DPYD‐overexpressing PDAC xenograft tumors revealed an upregulation of genes associated with metallopeptidase activity—MMP9 and MEP1A. Furthermore, the overexpression of MEP1A in PDAC was associated with invasion. Next, we investigated the combined effects of Lut, a DPYD suppressor, and 5‐FU on DPYD‐overexpressing xenograft tumors and PDAC of *Pdx1‐Cre*; *LSL‐KrasG12D/+*; *Trp53flox/flox(KPPC)* mice. Neither single administration of 5‐FU nor Lut showed significant inhibitory effects; however, the combined administration of 5‐FU and Lut exhibited a significant tumor‐suppressive effect in both the xenograft tumors and *KPPC* models.

**Conclusion:**

We have elucidated that DPYD expression contributes to proliferation, invasiveness, and 5‐FU resistance, in PDACs. The combination therapy of Lut and 5‐FU holds the potential for enhanced efficacy against PDACs.

## Graphical abstract

The heightened expression of dihydropyrimidine dehydrogenase (DPYD) in pancreatic cancer not only accelerates pyrimidine degradation but also stimulates cell proliferation and invasiveness, accompanied by the upregulation of MMP9 and MEP1A. A combination therapy comprising lutetolin, a DPYD inhibitor, and 5‐FU may be effective for treating pancreatic cancers.

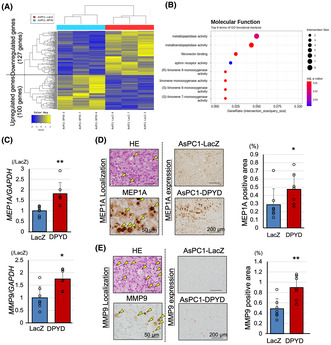

## 1 INTRODUCTION

Pancreatic cancer remains a lethal disease with a 5‐year survival rate of approximately 10%, despite advances in treatment that have improved the 5‐year survival rate for various type of cancer.^1^ In Japan, there were 43,865 new cases of pancreatic cancer reported in 2019, with 37,677 deaths due to pancreatic cancer in 2020.^2^ The limited effectiveness of newly developed immunotherapies and targeted therapies contributes to the high mortality rate in pancreatic cancer. Standard treatments in Japan mainly involve chemotherapy such as gemcitabine, the regimen tegafur, gimeracil, and oteracil (TS‐1), the regimen fluorouracil, leucovorin, irinotecan, and oxaliplatin (FOLFIRINOX), and the regimen gemcitabine and nab‐paclitaxel, however, their effectiveness is limited.

Our previous study demonstrated the inhibitory effect of luteolin (3′,4′,5,7‐tetrahydroxy flavone; Lut) on nitrosobis(2‐oxopropyl)amine‐induced pancreatic carcinogenesis in hamsters and its potential as a chemopreventive agent in pancreatic cancer. Lut is a flavonoid, specifically a flavone, found in celery, green pepper, parsley, and perilla leaf. Lut exhibits strong antioxidant activity and shows antiinflammatory and antitumor effects. Previous animal experiments have reported the anticancer effects of Lut in lung, colorectal, liver, ovarian, prostate, bladder and nonalcoholic steatohepatitis‐related liver cancers^3^, ^4^, ^5^, ^6^, ^7^ In the previous study, we had also reported that Lut decreases dihydropyrimidine dehydrogenase (DPYD) and phospoho‐STAT3 expression in pancreatic cancer cells as one of its mechanisms of activity.^8^ However, the detailed mechanism of how DPYD is involved in pancreatic cancer remains unclear.

DPYD is a catabolic enzyme involved in degrading pyrimidines such as 5‐FU, thymine, and uracil. 5‐FU, used in TS‐1 as a prodrug and in FOLFIRINOX therapy, exerts its anticancer effect by inhibiting RNA synthesis through 5FUTP and DNA synthesis via thymidine synthase inhibition. However, approximately 80% of the administered 5‐FU is degraded by DPYD into inactive substances.^9^ In TS‐1 therapy, DPYD is competitively inhibited by 5‐chloro‐2,4‐dihydroxypyrimidine, resulting in an increase in the blood concentration of 5‐FU. This concurrently reduces the neurotoxic and cardiotoxic side effects caused by the breakdown products of 5‐FU by DPYD.^10^ Additionally, some studies suggest that DPYD expression contributes to the epithelial‐mesenchymal transition in high‐grade cancer cell lines,^11^ although its role in pancreatic cancer remains unclear.

In this study, we extensively investigated the role of DPYD in pancreatic cancer using DPYD‐overexpressing pancreatic cancer cells and evaluated the therapeutic effect of a combination treatment with 5‐FU and Lut on pancreatic cancer using xenograft models and *Pdx‐Cre*; *LSL‐Kras* ^*G12D/+*^; *Trp53* ^*flox/flox*^ (*KPPC*) mice.

## 2 MATERIALS AND METHODS

Details of the Materials and Methods are given in Supplementary Materials and Methods, available at Cancer Medicine Online.

### 2.1 Cell culture and construction of DPYD‐ and MEP1A‐expressing vector

Human pancreatic cancer cell lines (AsPC1/RRID: CVCL_0152, PK1/RRID: CVCL_4717, PANC1/RRID: CVCL_0480, MIAPaCa‐2/RRID: CVCL_0428, KP4/RRID: CVCL_1338) were obtained from the American Type Culture Collection (ATCC, Rockville, MD), the Leibniz Institute DSMZ (Braunschweig, German), and gifted from Dr. Inaguma. They were maintained in RPMI1640 (Wako Pure Chemical Industries Co. Ltd., Osaka, Japan) supplemented with 10% fetal bovine serum (FBS). The cells were cultured at 37°C in humidified air containing 5% CO_2_. Cell authentication (STR profile) of all human cancer cell lines was performed using the GenePrint System (10 loci) performed from BEX Co. Ltd.

The method for constructing *DPYD‐* or *LacZ‐*overexpressing AsPC1, PK1, and PANC1 cell lines was previously described,^8^ and the same method was used for generating *MEP1A‐*overexpressing cells. In brief, the coding sequences of *DPYD* (NM_000110.4) and *MEP1A* (NM_005588) were generated by PCR using PrimeSTAR HS DNA Polymerase (Takara Bio, Shiga, Japan) with primers containing restriction enzyme sites (Table S1). The resulting *DPYD* and *MEP1A* sequences were then inserted into CSII‐CMV‐MCS‐IRES2‐Bsd vectors and transfected into AsPC1, PK1 and PANC1 cells, which were selected with blasticidin.

### 2.2 Xenograft model

Six‐week‐old KSN/Slc mice were purchased from Japan SLC, Inc. (Shizuoka, Japan) and acclimated to the animal facility for 1 week. They were maintained in plastic cages on hardwood chips, in an individually ventilated cage system at 22 ± 2°C and 50% humidity with 12 h/12 h light–dark cycle. All animal experiments were performed using protocols approved by the Institutional Animal Care and Use Committee of Nagoya City University Graduate School of Medical Sciences (No. IDO 20–027, IDO 20–051).

AsPC1/PK1‐LacZ and AsPC1/PK1‐DPYD cells (1.0 × 10^6^ cells) were subcutaneously implanted at 8 weeks into 7–8 mice of each group mice on the right and left sides of the dorsal surface. The tumor volumes (long diameter × short diameter × height × π/6) were measured for 8 weeks.

In the second experiment, AsPC1‐LacZ and AsPC1‐DPYD cells (1.0 × 10^6^ cells) were implanted in the same way. After 2 weeks, we randomized these mice into four groups: Control (*n* = 7), 5‐FU group (40 mg･kg^−1^･week^−1^, i.p., FujiFilm, Osaka, Japan; *n* = 7), Lut group (100 ppm in diet, Tokyo Chemical Industry Co., Ltd, Tokyo, Japan; *n* = 6), 5‐FU + Lut group (5‐FU: 40 mg･kg^−1^･week^−1^, Lut: 100 ppm; *n* = 7). All animals were fed the AIN76A powdered diet (OrientalBioService, Inc., Kyoto, Japan), and the Lut‐treated group received 100 ppm Lut mixed in the diet. The tumor volumes were measured for 9 weeks. When the mice were sacrificed, tumor weights were measured and the tumors were cut at the largest cross‐section; half of them were fixed in 10% neutral buffered formalin for histological analysis, and the other half was frozen. In HE staining, the area without enucleation or necrosis was defined as the viable area, and its area at the largest cross‐section was measured using CS2 Aperio. Additionally, TUNEL staining (Takara Bio Inc., Shiga, Japan) was performed, and the number of TUNEL‐negative cells, excluding diffuse positive necrotic areas and strongly stained dead or apoptotic cells, was measured using CS2 Aperio. Immunohistochemical staining was performed using antibodies against Ki67 (1:100, MIB1, Agilent Technologies, Ltd., CA, RRID:AB_2631211), DPYD (1:100, 7D4, Abcam plc, Cambridge, UK, RRID: AB_941338), MEP1A (1:1000, 364312, R&D Systems, MN, AB_2250624), MMP9 (1:500, EP1254, Abcam, RRID:AB_1310463), CD31 (1:50, Abcam, RRID:AB_726362), SMA (1:600, 1A4, Agilent, RRID:AB_2922801) with Leica Bond Max. The evaluation of DPYD staining was performed on six regions with strong staining intensity at 20× magnification, and the relative average brightness of the inverted black‐and‐white images was measured using the BZ‐X810 imaging system (KEYENCE, Osaka, Japan). The Ki‐67 proliferation index was measured using Aperio CS2, calculating the percentage of positive cells among more than 3000 cells in the hot spot. The areas of MMP9‐ and MEP1A‐positive staining within intraductal lesions were calculated as percentages of the total tumor area by the Aperio CS2 digital pathology slide scanner.

### 2.3 KPPC mouse model

The strains of *Pdx1‐Cre*; *LSL‐Kras* ^*G12D/+*^ and *Pdx1‐Cre*; *Trp53* ^*flox/+*^ mice that were used in previous studies^12^ were transferred from the Nihon University School of Medicine by the Jackson Laboratory. We crossed and generated the *Pdx1‐Cre*; *LSL‐Kras* ^*G12D/+*^; *Trp53* ^*flox/flox*^ (*KPPC*) mice. The produced mice underwent tail cutting at the fourth week, and DNA was extracted using the DirectPCR Lysis Reagent (Viagen Biotech, LA). PCR was performed using primers listed in Table S1. Five‐week‐old *KPPC* mice were treated with 5‐FU (40 mg･kg^−1^･week^−1^ i.p., *n* = 15), Lut (100 ppm in diet, *n* = 14), both 5‐FU and Lut (40 mg･kg^−1^･week^−1^ i.p., 100 ppm in diet, *n* = 15), or Control (*n* = 15) for 4 weeks. Lut was mixed in the diet, as in the transplanted tumor experiment. The body and pancreatic weights of the mice were measured at sacrifice. The tumor portion of the pancreas was partially frozen, while the remaining portion was divided into 2 mm‐wide sections along the short axis for histological evaluation. The tumor lesions in *KPPC* mice were identified as pancreatic cancer by two individuals, including a pathologist. If there was a difference in the two opinions, the final range was determined after discussion and comparison with other cases. The areas of the cancerous and non‐cancerous were assessed using Aperio CS2 on sections prepared by cutting the entire pancreas, excluding the frozen sections, at 2 mm intervals.

### 2.4 RNA‐seq analysis of xenograft tumors

We performed RNA‐seq using three samples each of AsPC1‐LacZ and AsPC1‐DPYD xenograft tumors. Gene expression analysis was performed using the TruSeq Standard Total RNA LT Sample Prep Kit (Macrogen Japan Corp, Japan) by following the manufacturer's instructions. To separate host and graft reads, the trimmed data were aligned to the Graft (GRCh38) and Host (mm10) references. Graft‐only reads were used to extract the gene expression profile for each sample. From the trimmed data containing 46,427 genes, we extracted 17,745 genes that were expressed in all samples (Data S1). Using the 227 genes that showed significant changes between AsPC1‐LacZ and AsPC1‐DPYD, we performed clustering and GO Enrichment Analysis. Detailed methods for the analysis are provided in the Supplementary Materials and Methods.

### 2.5 Tissue microarray analysis of human PDACs

We utilized our institution's computerized database to identify 132 patients diagnosed with pancreatic cancer who underwent surgery at the Department of Gastroenterological Surgery, Nagoya City University Hospital, between April 2004 and December 2019. Surgical specimens were fixed in 10% buffered formalin and subsequently embedded in paraffin. Two people (H.K., M.S) independently diagnosed all cases and selected two appropriate lesions from the paraffin blocks, with diameters of either 1.5 mm or 3 mm, for further analysis. We conducted the evaluation of DPYD immunohistochemical staining as previously described.^8^ Regarding MEP1A or MMP9 staining, if cytoplasmic or intraductal lesions exhibited strong positive cell staining, the cases were classified as the high‐expression group. The histological evaluation of immunostaining was initially conducted by two individuals (H.K., M.S) who reviewed several samples to establish the criteria. They then performed a blind assessment. In cases where evaluations differed, a discussion was held to reach a consensus by reassessing the samples in the context of the surrounding evaluations. This study was approved by the Institutional Review Board at Nagoya City University Graduate School of Medical Sciences (60‐19‐0115) and conducted in accordance with the guidelines of the Declaration of Helsinki.

### 2.6 Statistical analysis

Differences in the quantitative data, expressed as mean ± SD, between groups, were compared by one‐way ANOVA and Dunnett's post hoc test or unpaired/paired *T* test. The comparison of the survival curve was done by using the Grehan–Breslow–Wilcoxon test. All tests were analyzed by using Graph Pad Prism (GraphPad Software, Inc., La Jolla, CA, RRID: SCR_002798).

## 3 RESULTS

### 3.1 DPYD overexpression promotes PDAC progression

**FIGURE 1:**
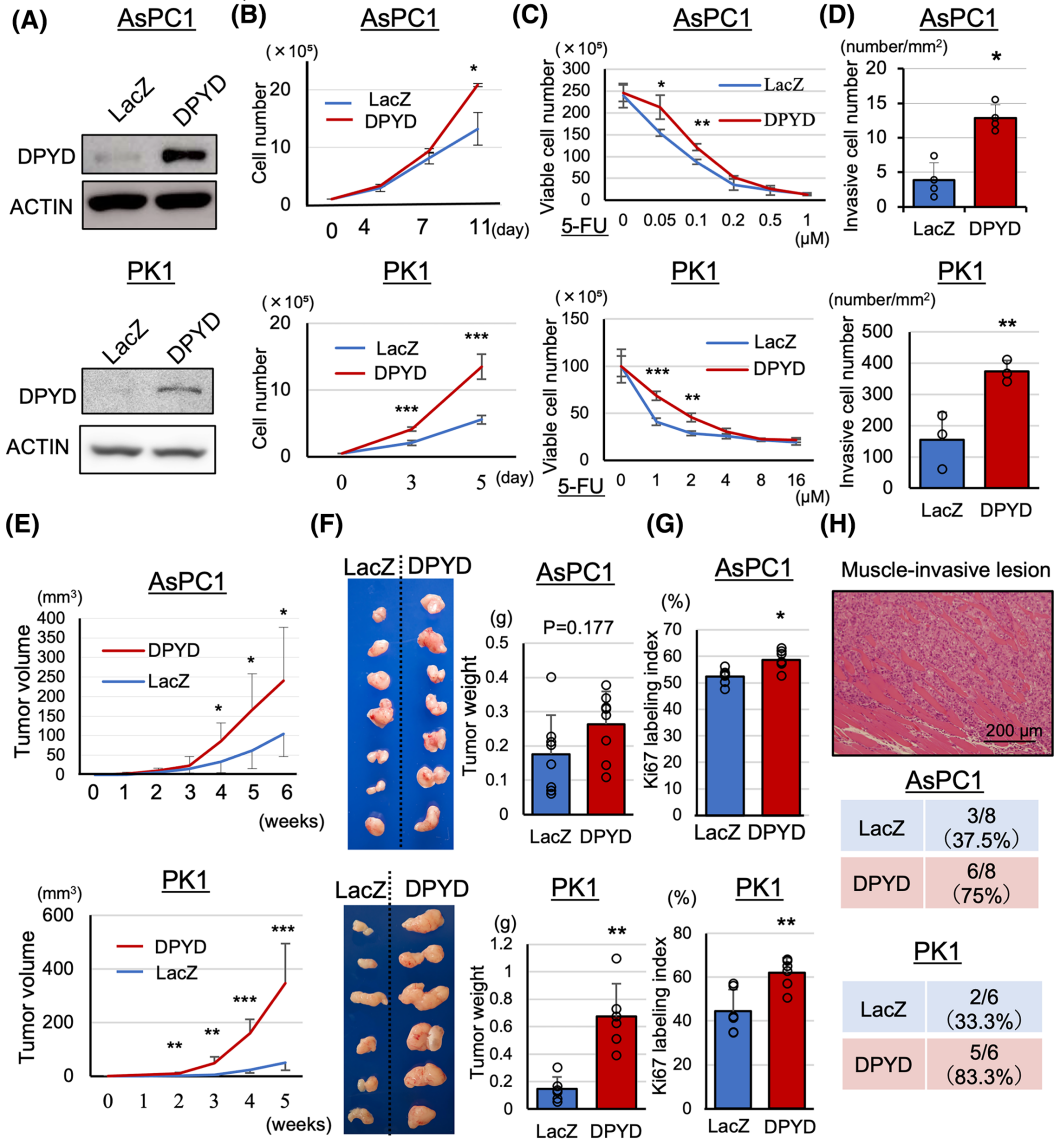
DPYD‐overexpressing PDAC cells show increased proliferation, invasiveness, angiogenesis, and 5‐FU resistance. AsPC1‐ and PK1‐DPYD cells overexpress protein (A) of DPYD. AsPC1‐ and PK1‐DPYD cells show increased proliferation (*n* = 4) (B), resistance to low doses of 5‐FU (*n* = 4) (C), and invasiveness by invasion assay (*n* = 3–4) (D) compared to AsPC1‐ and PK1‐LacZ cells in vitro. In the xenograft model, AsPC1‐ and PK1‐DPYD tumors show increased volume (E), weight (F), Ki67 labeling index (G), muscle invasion (H), compared to AsPC1‐ and PK1‐LacZ tumors (*n* = 8, 6). Data are represented as mean ± SD. *p < 0.05, **p < 0.01, ***p < 0.001 statistically significant compared with AsPC1‐ or PK1‐LacZ.

We had previously demonstrated that inhibition with *siDPYD* resulted in decreased proliferation in a high‐DPYD‐expressing pancreatic ductal adenocarcinoma (PDAC) cell line (MiaPaCaII cell line) in vitro.^8^ However, since we had not examined the effects of overexpression on pancreatic cancer cells or performed in vivo studies, we overexpressed DPYD in low‐DPYD‐expressing cells and investigated its impact on proliferation and invasiveness both in vitro and in vivo. The expression of DPYD mRNA and protein was increased in AsPC1, PK1, and PANC1 cells (low‐DPYD‐expressing PDAC cell lines, Figure S1A) transfected with the CSII‐CMV‐MCS‐IRES2‐Bds vector containing DPYD compared to the vector containing LacZ as shown in Figure 1A and Figure S1B,C. The DPYD‐overexpressing cells demonstrated increased cell growth measured by cell counts and the WST‐1 assay (Figure 1B; Figure S1D,E), increased chemoresistance to low doses of 5‐FU measured by cell counts and the WST‐1 assay (Figure 1C, Figure S1F), and enhanced invasiveness measured by an invasion assay as compared to LacZ‐induced cells (Figure 1D; Figure S1G). To investigate tumor initiation, AsPC1‐LacZ and ‐DPYD cells were subcutaneously transplanted at densities of 10,^3^ 10^4^, 10^5^, and 10^6^ cells (*n* = 7–8) to determine their tumor‐forming ability.^13^ However, no significant difference was observed in the tumor‐initiating cell frequency between AsPC1‐LacZ (1/14,754) and AsPC1‐DPYD (1/10,671) cells (Figure S2A). In a xenograft model, the tumors derived from DPYD‐overexpressing cells had a larger volume and weight than those from control cells (Figure 1E,F). Additionally, Ki67 staining demonstrated a significant increase in proliferation (Figure 1G, Figure S2B), and the number of invasion tumors into muscles tended to increase with DPYD‐overexpressing cells compared to the control cells (Figure 1H). The CD31 staining showed significantly elevated angiogenesis in AsPC1‐DPYD tumors compared to the AsPC1‐LacZ tumors (Figure S2C), but the evaluation of tumor angiogenesis in PK1 cells was difficult because of squamous differentiation. The αSMA‐positive intratumoral stroma in AsPC1‐DPYD xenograft tumors was significantly increased compared to AsPC1‐LacZ (Figure S2D). These in vitro and xenograft model results indicated that high DPYD expression promoted proliferation and invasiveness, increased angiogenesis and intratumoral stroma, and enhanced resistance to 5‐FU.

### 3.2 Overexpressed DPYD accelerated metallopeptidase‐related mRNAs and proteins

**FIGURE 2:**
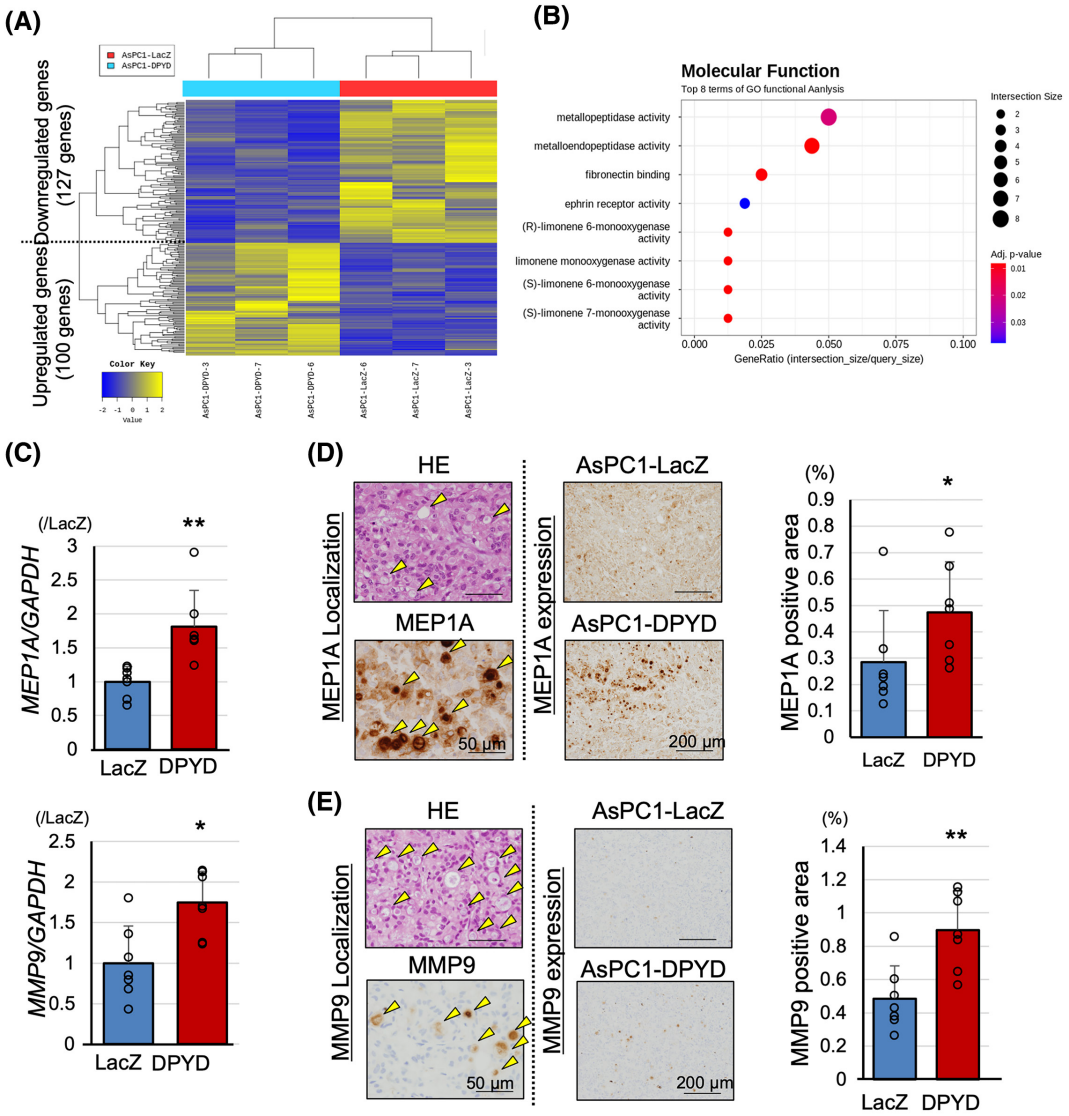
DPYD‐overexpressing PDAC cells show enhanced metallopeptidase expression. Heat map of RNA‐seq on AsPC1‐DPYD (*n* = 3) and AsPC1‐LacZ xenograft tumors (*n* = 3) (A), and its Gene Ontology analysis with respect to molecular function (B). AsPC1‐DPYD xenograft tumors (*n* = 7) show upregulation of genes associated with metallopeptidase activity, including MEP1A and MMP9 (C). Immunohistochemical analysis of MEP1A (D) and MMP9 (E) indicate that these proteins are secreted into intraductal lesions (yellow arrowheads) and are upregulated in AsPC1‐DPYD compared to AsPC1‐LacZ xenograft tumors (*n* = 8). Data are represented as mean ± SD. *p < 0.05, **p < 0.01, statistically significant compared with AsPC1‐LacZ.

To investigate the detailed mechanisms through which DPYD contributes to cancer progression, RNA‐seq analysis was conducted using three xenograft tumors each of AsPC1‐DPYD and AsPC1‐LacZ. Utilizing multidimensional scaling analysis, we were able to detect differential expression between the AsPC1‐DPYD and AsPC1‐LacZ groups (Figure S3A). In Figure 2A, the results revealed that 100 genes exhibited a *Z*‐score increase of over twofold, while 127 genes exhibited a *Z*‐score decrease of half or less in AsPC1‐DPYD tumors compared to AsPC1‐LacZ tumors; the top 20 genes showing altered expression are listed in Table S2. Molecular function‐based Gene Ontology analysis highlighted a significant alteration in the genes associated with metallopeptidase and metalloendopeptidase activities (Figure 2B). Specifically, the upregulated genes within the metallopeptidase activity ontology, *MMP9* and *MEP1A*, exhibited significantly elevated mRNA levels in AsPC1‐DPYD tumors relative to AsPC1‐LacZ tumors (Figure 2C). Moreover, immunohistochemical staining demonstrated MEP1A and MMP9 protein expression within the tumor gland ducts of the xenograft tumors, with a notably higher percentage of the positively stained area observed in AsPC1‐DPYD tumors than in AsPC1‐LacZ tumors (Figure 2D,E). To exclude nonspecific reactions of the MMP9 antibody, we included (i) a negative control using an isotype control and carried out staining with only the secondary antibody and (ii) a positive control using myeloid tissue (Figure S3C). In the subcutaneous xenograft tumors of PK1‐DPYD, the mRNA levels of *MMP9* and *MEP1A* were also increased, and there was a significant increase in the number of MMP9‐positive areas in immunohistochemical staining compared to the PK1‐LacZ tumors (Figure S3B,D). These findings suggest the involvement of DPYD in metallopeptidase activity, particularly in the secretion of MMP9 and MEP1A.

### 3.3 MEP1A expression promoted invasiveness

**FIGURE 3:**
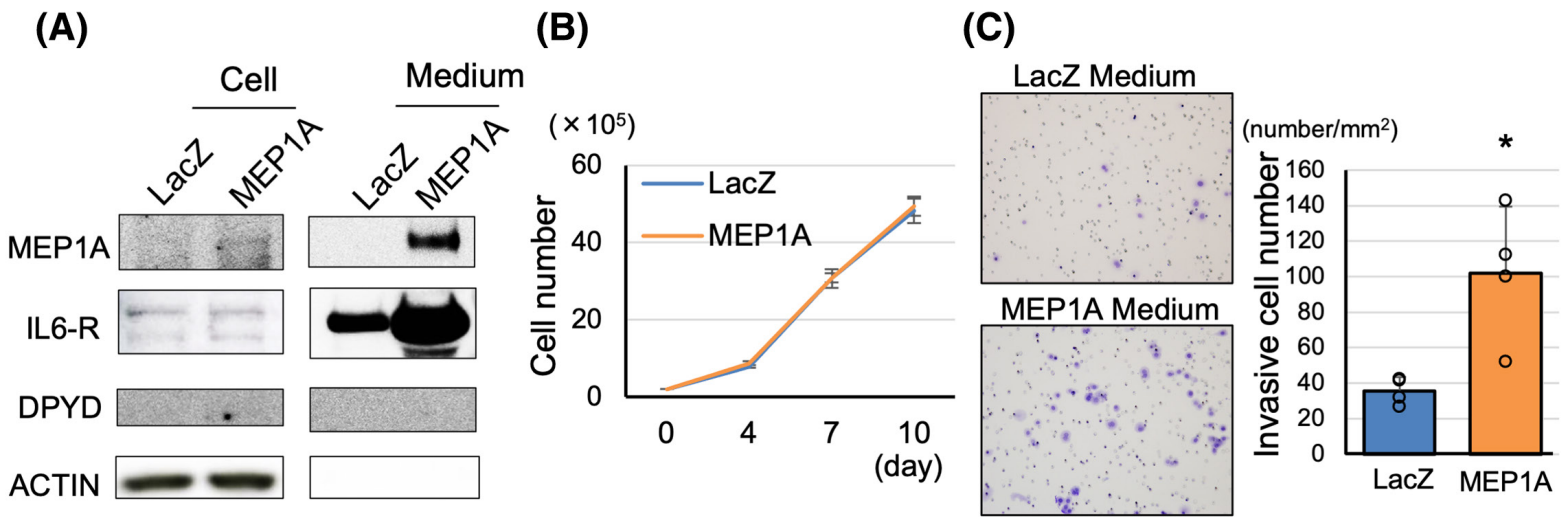
MEP1A overexpression in PDAC cells does not affect proliferation but promotes invasiveness. MEP1A‐overexpressing AsPC1 cells secrete MEP1A into the medium (A). MEP1A expression does not contribute to proliferation (*n* = 4) (B) but promotes invasiveness (*n* = 4) (C). Data are represented as mean ± SD. *p < 0.05 statistically significant compared with AsPC1‐LacZ.

Despite the established importance of MMP9 in mediating invasiveness and metastasis in pancreatic cancer,^14^ the role of MEP1A in PDACs remains unclear. To gain insight into this issue, we generated MEP1A‐overexpressing AsPC1, PK1, and PANC1 cells by using the CSII‐CMV‐MCS‐IRES2‐Bds vector (Figure S4A). In the MEP1A‐overexpressing cells, whereas the protein levels of MEP1A and its shedding target, IL6‐R, were minimal upregulated in the cultured cells, they were increased in the culture medium, and there was no change in DPYD expression due to the upregulation of MEP1A (Figure 3A, Figure S4B). Proliferation measured by cell counts and the WST‐1 assay was not altered in MEP1A‐overexpressing cells compared to the control cells (Figure 3B; Figure S4C,D), although invasiveness was enhanced when AsPC1 cells were treated with the supernatant medium of the MEP1A‐overexpressing cells medium was treated compared to the control medium (Figure 3C, Figure S4E). These results suggested that the increased expression of MEP1A, induced by DPYD expression, leads to MEP1A secretion into the extracellular medium, affecting cell invasiveness but not cell proliferation.

### 3.4 The combined therapy of 5‐FU and luteolin suppressed xenograft tumors

**FIGURE 4:**
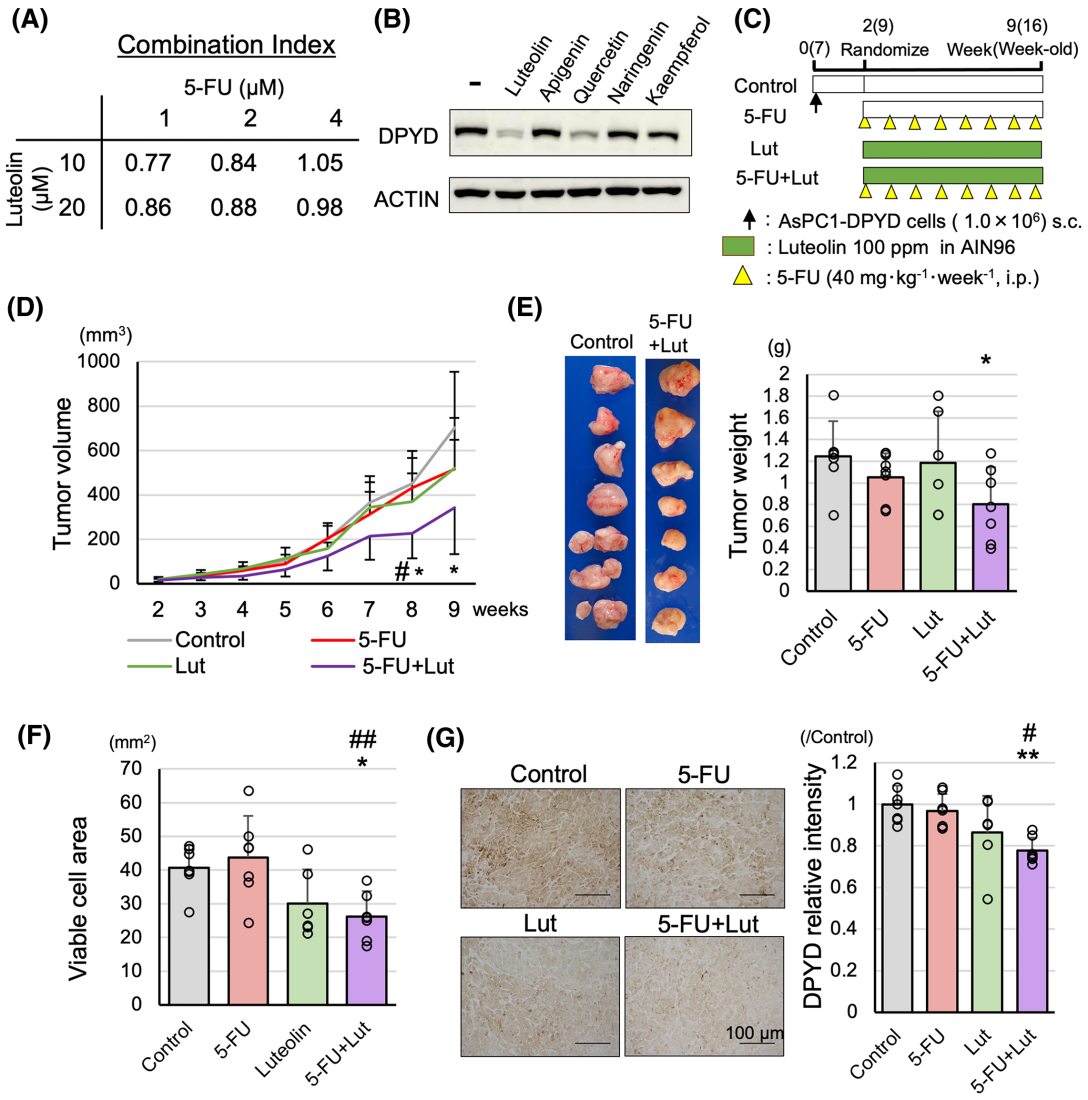
Effect of combination therapy of 5‐FU and Luteolin on AsPC1‐DPYD xenograft tumors. Combination index of 5‐FU and Lut in KP4 cells (*n* = 3) (A). DPYD inhibitory effects of Lut and other flavonoids in KP4 cells (B). The schematic of treatment for AsPC1‐DPYD xenograft tumors (C), and combination treatment of 5‐FU and Lut significantly decreased tumor volume (D) and weights (E) compared to untreated control (*n* = 6–7). Viable cell areas of tumors on HE staining (F) and DPYD relative intensity to control (G) are significantly decreased in 5‐FU and Lut groups compared to the 5‐FU group and the control group (*n* = 6–7). Data are represented as mean ± SD. *p < 0.05, **p < 0.01, as compared to the control group. ^#^ *p* < 0.05, ^##^ *p* < 0.01, as compared to the 5‐FU group.

DPYD has been shown to not only degrade 5‐FU but also contribute to proliferation, invasiveness, and angiogenesis in pancreatic cancer. As Lut suppresses DPYD protein expression^8^ (Figure S5A), we investigated the combined effects of 5‐FU and Lut in pancreatic cancer. Using a graph for the growth inhibition by Lut (0–60 μM) or 5‐FU (0–32 μM) as monotherapy on the KP4 cells (Figure S5B), we calculated the combination index (CI) by using the Chou‐Talalay Method^15^ when Lut (10, 20 μM) and 5‐FU (1, 2, 4 μM) were combined. As shown in Figure 4A, the CI suggested a synergic effect of 5‐FU and Lut, especially in low doses. To investigate whether this DPYD inhibitory effect was specific to Lut, the DPYD inhibitory effects of several flavonoids were examined. We treated KP4 cells (high DPYD‐expressing PDAC cell line) (Figure S1A) with 25 μM of each compound, including flavonoids such as apigenin (flavone), quercetin(flavonol), kaempferol (flavonol), and naringenin (flavanone), for 48 h. DPYD inhibition was not observed with apigenin, which lacks a hydroxy group at the 3′ position despite having the same flavone structure. In contrast, quercetin, which has a flavanol scaffold and a hydroxy group at the same position, exhibited an inhibitory effect on DPYD expression. This suggests that the 3′,4′‐ortho‐diphenol scaffold may be crucial for the DPYD inhibitory effect of Lut (Figure 4B; Figure S4C).

Next, we subcutaneously transplanted AsPC1‐DPYD cells and treated nude mice for 7 weeks as shown in Figure 4C. There was no significant change in food intake among the groups (Figure S5D). The results indicated that while single treatments with Lut or 5‐FU did not show any significant inhibitory effects on tumor volume and tumor weight, the combination treatment of 5‐FU and Lut exhibited a significant tumor‐suppressive effect compared to the untreated control group (Figure 4D,E). Additionally, the viable tumor cell area in the largest cross‐section of HE and TUNEL staining was significantly reduced in the Lut and 5‐FU combination treatment group compared to the control group (Figure 4F, Figure S5E). The relative intensity of DPYD was significantly reduced in the Lut and 5‐FU combination treatment group compared to the control group and the 5‐FU single‐treatment group (Figure 4G). However, no values indicating a synergistic effect were calculated in a xenograft model. Thus, the combined administration of Lut and 5‐FU could be effective in the treatment of pancreatic cancer.

### 3.5 The combined therapy of 5‐FU and luteolin suppressed pancreatic cancer in KPPC mice

**FIGURE 5:**
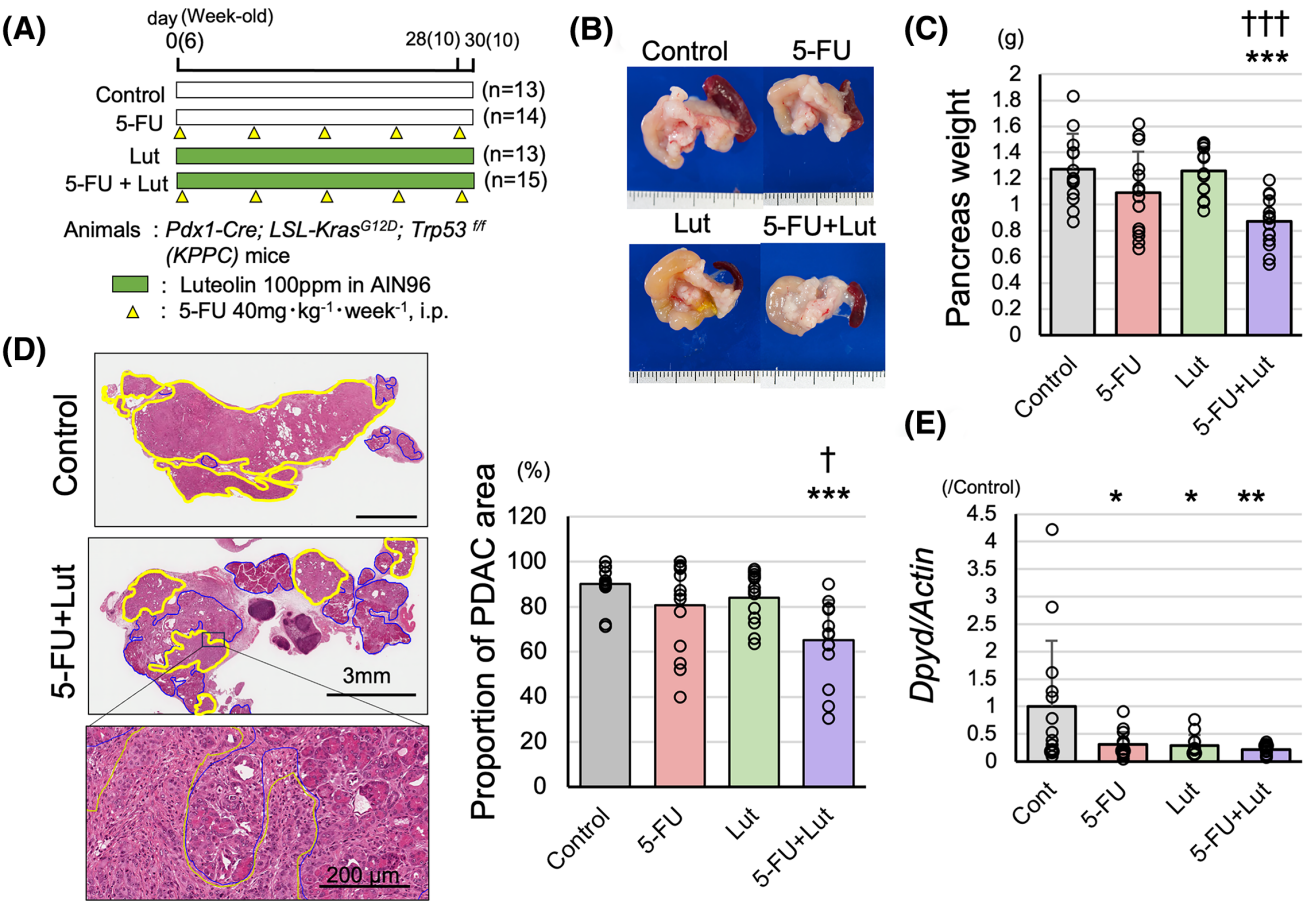
Effect of combination therapy of 5‐FU and Luteolin effect on KPPC mice. The schematic of treatment for PDACs of KPPC mice (A). Combination therapy of 5‐FU and Lut significantly decreased pancreas weights (B, C) and the proportion of PDAC in the pancreatic sections (D) compared to untreated control and Lut single‐treatment (yellow circle: PDAC area, blue circle: non‐cancerous lesion) (*n* = 13–15). *DPYD* mRNA levels are decreased in 5‐FU, Lut, and 5‐FU + Lut groups compared to the control group (*n* = 13–15) (E). Data are represented as mean ± SD. *p < 0.05, **p < 0.01, ***p < 0.001 as compared to the control group. ^†^ *p* < 0.05, ^†††^ *p* < 0.001, as compared to the Lut group.

We used *KPPC* mice to confirm the effects of combination treatments of 5‐FU and Lut in a more clinically relevant model. The experimental protocol involved a 4‐week treatment period, as shown in Figure 5A. During the treatment period, 7 out of 13 mice in the control group died due to PDAC, and the survival curve showed a tendency for poorer prognosis compared to the treatment groups (Figure S6A). At the time of sacrifice, all mice had developed pancreatic cancer, with a significant replacement of pancreatic tissue by PDACs (Figure 5B). The pancreatic weight was significantly decreased in the combination treatment group of 5‐FU and Lut compared to the control group and the Lut monotherapy group (Figure 5C). Regarding the proportion of PDAC area in the whole pancreatic section of H&E staining, although no significant therapeutic effect was observed in the monotherapy groups, the combination treatment group of 5‐FU and Lut showed a significant decrease compared to the control group (Figure 5D), and *Dpyd* mRNA levels were decreased in the combination treatment group compared to the control group (Figure 5E). Thus, the combination treatment of 5‐FU and Lut in *KPPC* mice also indicated the potential usefulness for pancreatic cancer treatment.

### 3.6 Clinicopathological features of DPYD and MEP1A expression in human PDAC tissue array

**FIGURE 6:**
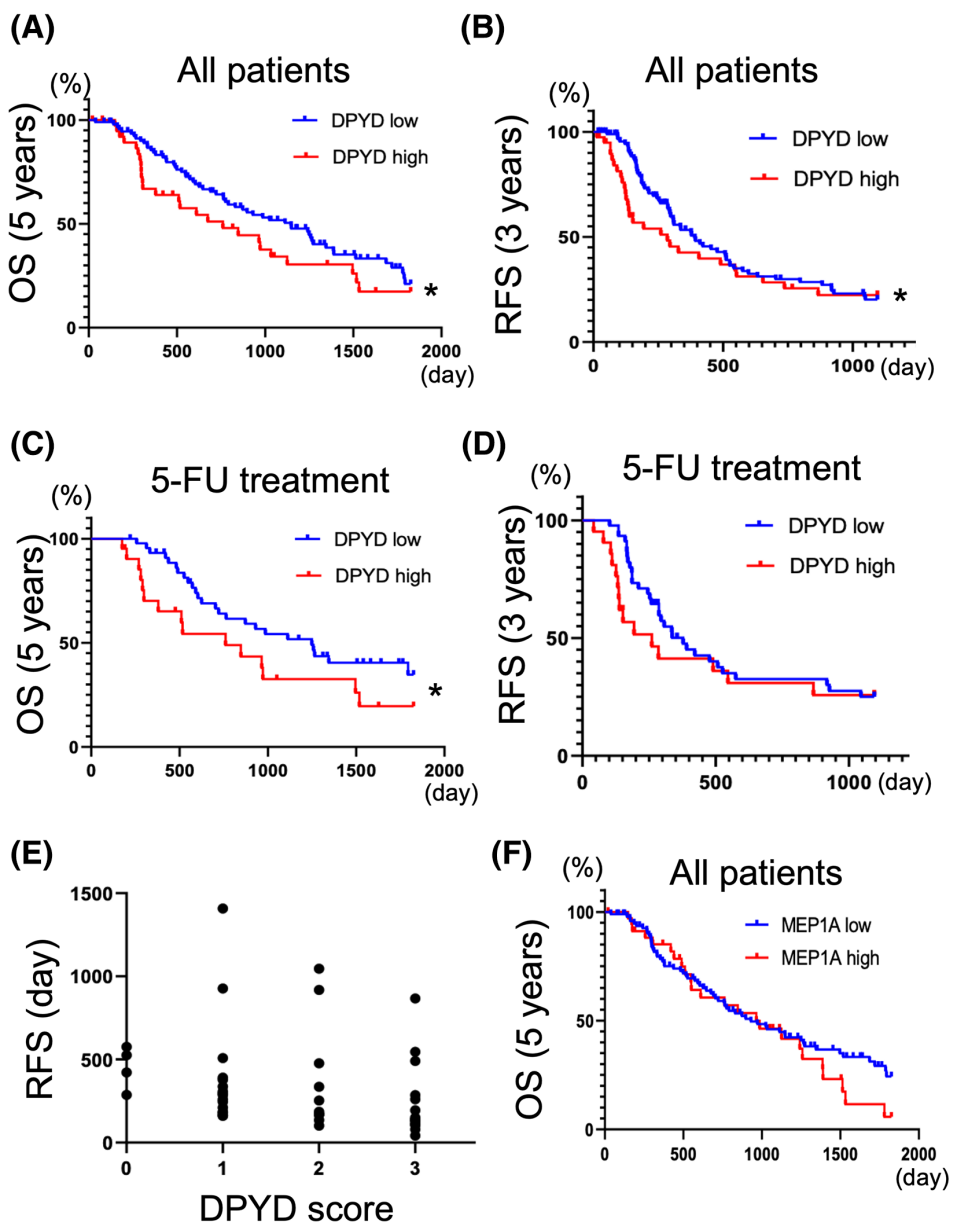
DPYD expression in human PDAC tissue microarray is associated with poor prognosis. Five‐year overall survival (OS) (A) and 3‐year recurrence‐free survival (RFS) (B) of PDAC patients with low‐DPYD (*n* = 91) and high‐DPYD expression (*n* = 41). Five‐year overall OS (C) and three‐year RFS (D) on PDAC patients with 5‐FU chemo adjuvant treatment, classified into low‐DPYD (*n* = 45), and high‐DPYD (*n* = 21) PDAC groups. The graph of recurrence‐free duration versus DPYD expression score in PDAC patients who received adjuvant 5‐FU chemotherapy but experienced PDAC recurrence (*n* = 49) (Spearman *p* < 0.01, *r* = −0.4365) (E). Five years (OS) in PDAC patients classified into MEP1A low (*n* = 97) and MEP1A high (*n* = 35) groups (F). *p < 0.05 statistically significant difference compared by using the Grehan–Breslow–Wilcoxon test.

In our previous paper, we reported that the expression of DPYD in human pancreatic cancer tissue arrays is a poor prognostic factor.^8^ In this study, we increased the sample size, conducted a more detailed analysis to investigate the relationship with 5‐FU treatment, and performed a histopathological analysis of MEP1A expression. The expression of DPYD was evaluated as in the previous paper, with 41 patients classified as DPYD high (DPYD score 3+) and 91 patients classified as DPYD low (DPYD score 0–2; Figure S7A). The 5‐year overall survival rates (OS) and 3‐year recurrence‐free survival rates (RFS) were significantly worse in DPYD high patients than in DPYD low patients (Gehan–Breslow–Wilcoxon test: *p* = 0.0484, 0.0346) (Figure 6A,B), and DPYD high patients had tumors with poorer differentiation (Table S3). From all patients, we extracted patients (*n* = 66) who received neoadjuvant 5‐FU chemotherapy, including TS‐1 and FOLFIRINOX, for at least 1 month (DPYD high/low: *n* = 21/45). The 5‐year OS was also significantly worse in DPYD high patients than in DPYD low patients, and the trend of the difference was more pronounced when compared across all patients (Gehan–Breslow–Wilcoxon test: *p* = 0.0308) (Figure 6C). A similar trend was observed in the 3‐year RFS rates of patients with 5‐FU treatment, but there was no significant difference due to insufficient sample size (Figure 6D). In addition, among the patients who experienced recurrence (*n* = 49), a significant negative correlation was observed between the DPYD expression score (scores 0–2: DPYD low, score 3: DPYD high) and the time to recurrence (Spearman *p* < 0.01, *r* = −0.4365) (Figure 6E). The expression of MEP1A was detected in 132 patients—35 patients were classified as MEP1A high and 97 patients as MEP1A low (Figure S7B). No significant difference was observed in the prognosis analysis (OS, RFS) between the two groups (Figure 6F, Figure S7C), however, in the histopathological analysis, MEP1A high patients had significantly poorer differentiation and larger tumors than the MEP1A low patients. Furthermore, a correlation was observed between MEP1A expression and DPYD expression (Table 1). These results indicated that pancreatic cancer with high expression of DPYD exhibited poor prognosis and poorer differentiation; similar results were obtained when focusing on the 5‐FU treatment group. The expression of MEP1A showed correlations with DPYD expression, differentiation, and size of tumors, but had no impact on prognosis.

**TABLE 1:** Clinicopathological characteristics of MEP1A high or low pancreatic ductal carcinoma.

	MEP1A		*p* value
	Low	High	
*n*	97	35	
Male/female	64/33	24/11	
Age (median)	69	70	
Differentiation			
Well	51 (53%)	6 (17%)	<0.001
Moderately	28 (29%)	11 (31%)	
Poorly	18 (19%)	18 (51%)	
Size (mm)			
0–20	22 (23%)	6 (17%)	<0.05
21–40	58 (60%)	17 (49%)	
41–60	15 (15%)	10 (29%)	
>61	2 (2%)	2 (6%)	
N (+)	61 (63%)	22 (63%)	
M (+)	2 (2%)	1 (3%)	
Resection margin	14 (14%)	9 (26%)	
DPYD			
0	9 (9%)	1 (3%)	
1+	44 (45%)	10 (29%)	
2+	21 (22%)	8 (23%)	
3+	23 (24%)	16 (46%)	
DPYD score	1.60	2.11	<0.01

## 4 DISCUSSION

In the present study, we have demonstrated that the upregulation of DPYD expression in pancreatic cancer is associated with proliferation, invasiveness, and angiogenesis as well as resistance to 5‐FU treatment. The increased expression of DPYD in PDACs contributes to the upregulation of genes expression involved in metallopeptidase activity such as MMP9 and MEP1A, which promoted the invasiveness of PDACs.

MMP9, classified as a gelatinase along with MMP2, has been reported to be involved in the invasiveness of various cancers, including PDACs, through the degradation of many extracellular matrix proteins^16^, ^17^ Increased expression of MMP9 has been reported in pancreatic cancer compared to normal pancreatic tissue, with the expression increasing with disease progression to PDACs in *Pdx1‐Cre*; *LSL‐Kras* ^*G12D/+*^; *Trp53* ^*flox/+*^ mice. The inhibition of *MMP9* expression by shRNA decreased cell proliferation, migration, and invasiveness in vitro.^14^ Another study showed that MMP9‐overexpressing PDAC cells exhibited higher levels of proliferation and angiogenesis than the control.^18^ However, the correlation between MMP9 expression and clinicopathological factors in pancreatic cancer patients remains inconclusive. Some studies have suggested a correlation of MMP9 expression with poor prognosis or lymph node metastasis,^19^ while others have reported no clear correlation with the prognosis or clinicopathological factors of PDACs.^20^ In our study, although a correlation was observed between DPYD expression and MMP9 in PDAC tissue arrays, no correlation of MMP9 expression was found with differentiation, size, lymph node metastasis, OS, or RFS (Figure S7D,E, Table S4). However, as MMP9 is a secreted protein, the generated protein may be quickly secreted as observed in our subcutaneous xenograft tumors of AsPC1‐DPYD (Figure 2E). Conversely, it is also possible to observe a positive signal in the cytoplasm, as seen in PK1‐DPYD cells (Figure S3D). Therefore, tissue‐based evaluations must be complemented with other assays to get a clearer picture. In fact, the proteomic analysis of pancreatic fluid of PDAC patients has shown increased MMP9 expression compared to normal pancreas.^21^ These results suggest that increased MMP9 expression is important for the progression of PDAC; however, its relationship with poor prognosis remains controversial.

However, there are few reports on MEP1A. In pancreatic cancer, MEP1A levels have been reported to significantly increase in the urine of patients with PDACs compared to that with chronic pancreatitis, and its expression has been associated with angiogenesis.^22^ MEP1A has been identified as a poor prognostic factor in hepatocellular carcinoma and has been reported to be involved in invasion and metastasis.^23^ Although no correlation with prognosis was observed in our study, MEP1A showed correlation with tumor size, differentiation, and DPYD expression. Further, AsPC1 with high MEP1A expression demonstrated enhanced invasiveness. The role of meprin, consisting of MEP1A or MEP1B, in tumors is known to include the extracellular matrix remodeling, pro‐angiogenic effects through VEGF‐A cleavage, and the activation of cytokine shedding such as IL‐6R.^24^, ^25^ In fact, the culture medium of AsPC1 with high MEP1A expression showed an increase in IL‐6R expression. These results suggest that the enhanced invasiveness due to high MEP1A expression may involve signaling pathways such as IL6R–IL6–STAT3. Furthermore, we had previously demonstrated that Lut inhibits the expression of STAT3 signaling and DPYD, and that there is a mutual dependency between STAT3 signaling and DPYD expression.^8^ These findings suggest the potential involvement of the DPYD–MEP1A–STAT3 pathway; however, further research is required to confirm this.

There are limitations to this study. It remains unclear how the upregulation of *DPYD* regulates metallopeptidase activity. Shaul et al. demonstrated the importance of DPYD expression in EMT using mammary epithelial cells. These researchers showed that reduced enzyme activity of DPYD contributed to the downregulation of EMT‐related genes, and the accumulation of dihydropyrimidines (DHPs), the metabolites, was crucial.^11^ In our study, it is unclear whether the enzyme activity of DPYD is important or if other functions are involved, emphasizing the need for further research. Another point to consider is how DPYD expression affects the tumor environment. This study focused on the differences in DPYD expression in PDAC cells and their impact on cell proliferation and invasion. However, human PDAC tissue consists of a complex tumor environment including extensive fibrosis. In our experiments, we observed increased angiogenesis and proliferation of αSMA‐positive cells in high‐DPYD‐expressing pancreatic cancer cells within xenograft tumors (Figure S2D,E). These results indicate that DPYD expression in PDAC cells may influence the tumor environment.

In this study, we observed the therapeutic effects of the combined treatment with 5‐FU and Lut, an inhibitor of DPYD, in PDACs. The combined treatment resulted in remarkable therapeutic effects, whereas 5‐FU or luteolin alone showed no significant effects. A synergistic effect of the combination treatment was observed in vitro. Previous studies have investigated the combined treatment of Lut and Gemcitabine in a xenograft model using BxPC3 cells.^26^ In that experiment, significant inhibition was observed only with the combination treatment, not with monotherapy. In our study, the effects of the combined treatment were represented by a polynomial model in generalized linear regression, with partial regression coefficients of −0.183 [5‐FU], −0.015 [Lut], and −0.204 [5FU + Lut] for the impact on pancreatic weight in *KPPC* mice. Therefore, although the combined treatment had the greatest impact, none of the coefficients were statistically significant due to which the synergistic effect could not be clearly defined. There are several possible reasons for this; one reason is that the treatment with 5‐FU or Lut alone could only be administered within an inadequate therapeutic range of concentrations. Another reason could be the weak expression of DPYD in *KPPC* mice with pancreatic cancer. Therefore, further detailed investigation is necessary to assess the impact on pancreatic cancer with high DPYD expressing PDACs and effective treatment doses of 5‐FU and Lut.

In conclusion, the heightened expression of DPYD in PDACs not only accelerates pyrimidine degradation but also stimulates cell proliferation and invasiveness, accompanied by the upregulation of MMP9 and MEP1A. A combination therapy comprising Lut, a DPYD inhibitor containing a 3′,4′‐ortho‐diphenol structure, and 5‐FU may be effective for treating PDACs.

## AUTHOR CONTRIBUTIONS

**Hiroyuki Kato:** Conceptualization (equal); data curation (equal); formal analysis (equal); investigation (equal); methodology (equal); project administration (equal); visualization (equal); writing – original draft (equal). **Motonori Sato:** Formal analysis (equal); investigation (equal); visualization (equal). **Aya Naiki‐Ito:** Conceptualization (equal); supervision (equal); writing – review and editing (equal). **Shingo Inaguma:** Resources (equal); supervision (equal). **Makoto Sano:** Resources (equal); writing – review and editing (equal). **Masayuki Komura:** Investigation (equal). **Yuko Nagayasu:** Investigation (equal). **Kuang Xiaochen:** Investigation (equal). **Akihisa Kato:** Resources (equal). **Yoichi Matsuo:** Resources (equal). **Hideaki Ijichi:** Resources (equal); writing – review and editing (equal). **Satoru Takahashi:** Supervision (equal); writing – review and editing (equal).

## CONFLICT OF INTEREST STATEMENT

There are no potential conflicts of interest to declare.

## ETHICS STATEMENT

Approval of the research protocol by an Institutional Reviewer Board and Informed Consent: Nagoya City University Graduate School of Medical Sciences (60‐19‐0115). Registry and the Registration No. of the study/trial: N/A. Animal Studies: the Institutional Animal Care and Use Committee of Nagoya City University Graduate School of Medical Sciences (No. IDO 20–027, IDO 20–051).

## Supporting information


Figure S1.

Figure S2.

Figure S3.

Figure S4.

Figure S5.

Figure S6.

Figure S7.

Table S1.

Table S2.

Table S3.

Table S4.

Data S1.

Supplementary Materials and Methods.

## Data Availability

The authors confirm that the data supporting the findings of this study are available within the article and its supplementary materials.
