# Supplementary figures and images for: The role of DPYD and the effects of DPYD suppressor luteolin combined with 5‐FU in pancreatic cancer
Source: Cancer Med. 2024 Aug 19;13(16):e70124. doi: 10.1002/cam4.70124 (PMC11331593; doi:10.1002/cam4.70124)

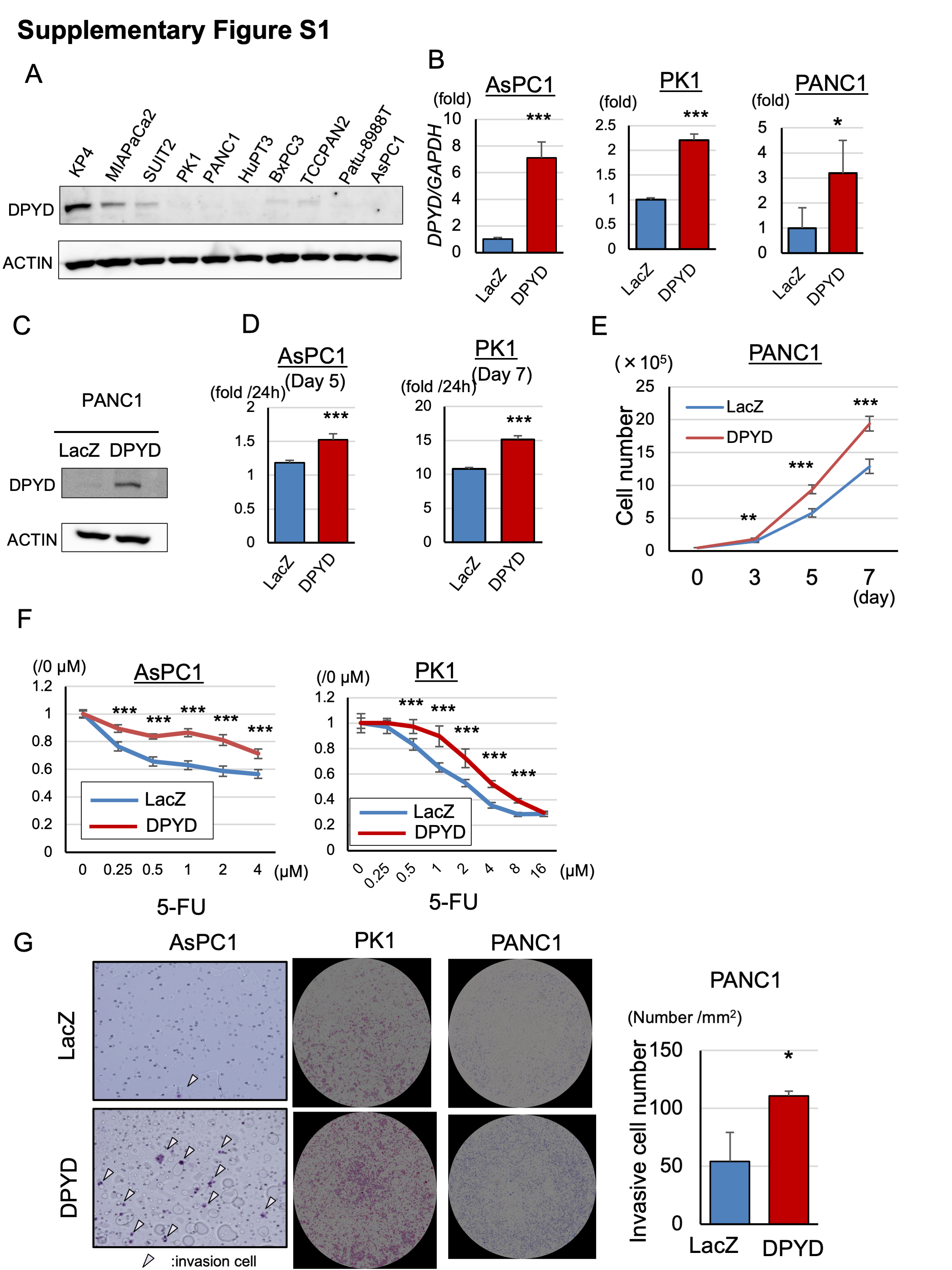

Supplement: Supplementary file 1 — Figure S1. [file CAM4-13-e70124-s005.tiff]

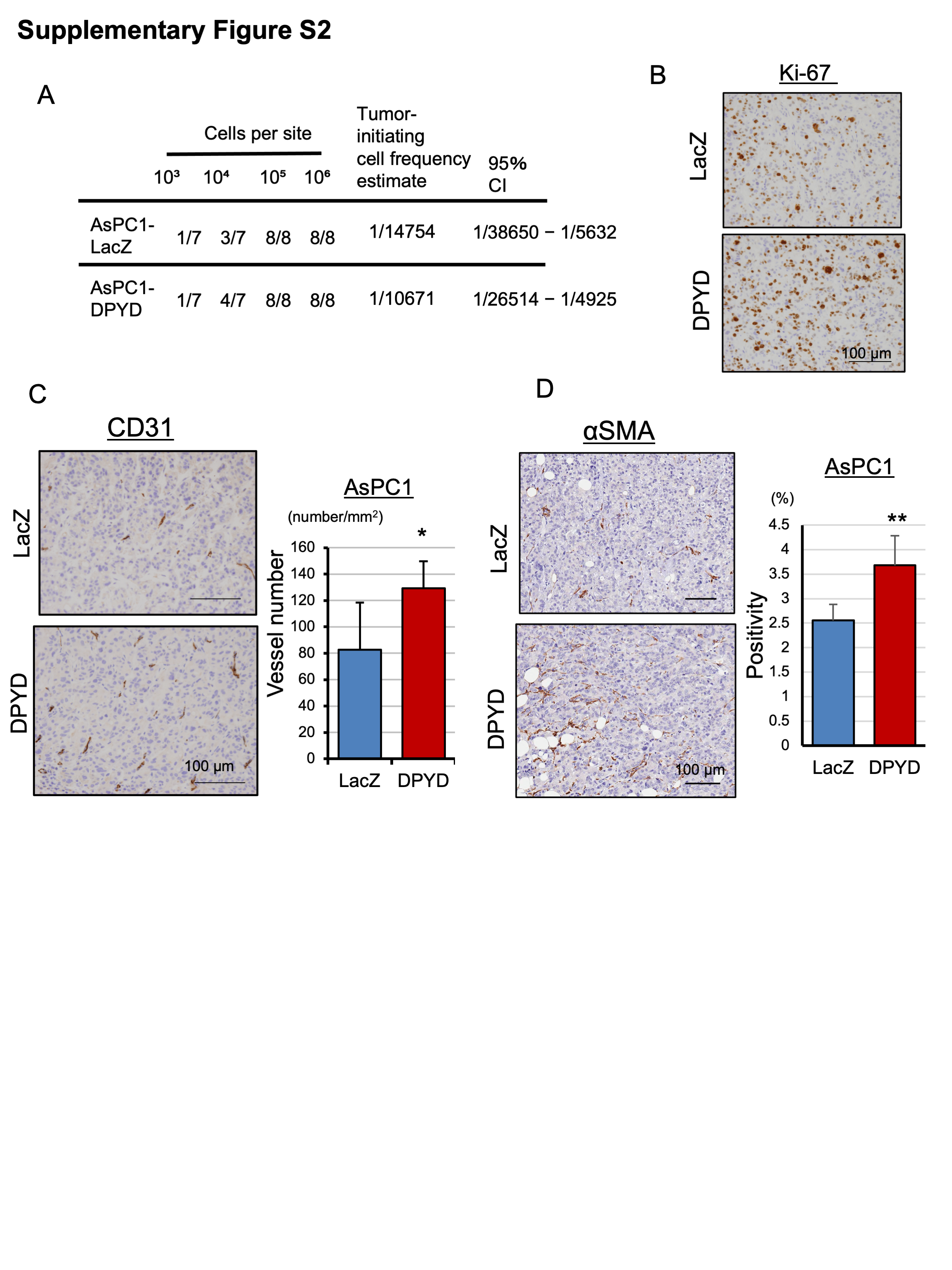

Supplement: Supplementary file 2 — Figure S2. [file CAM4-13-e70124-s008.tiff]

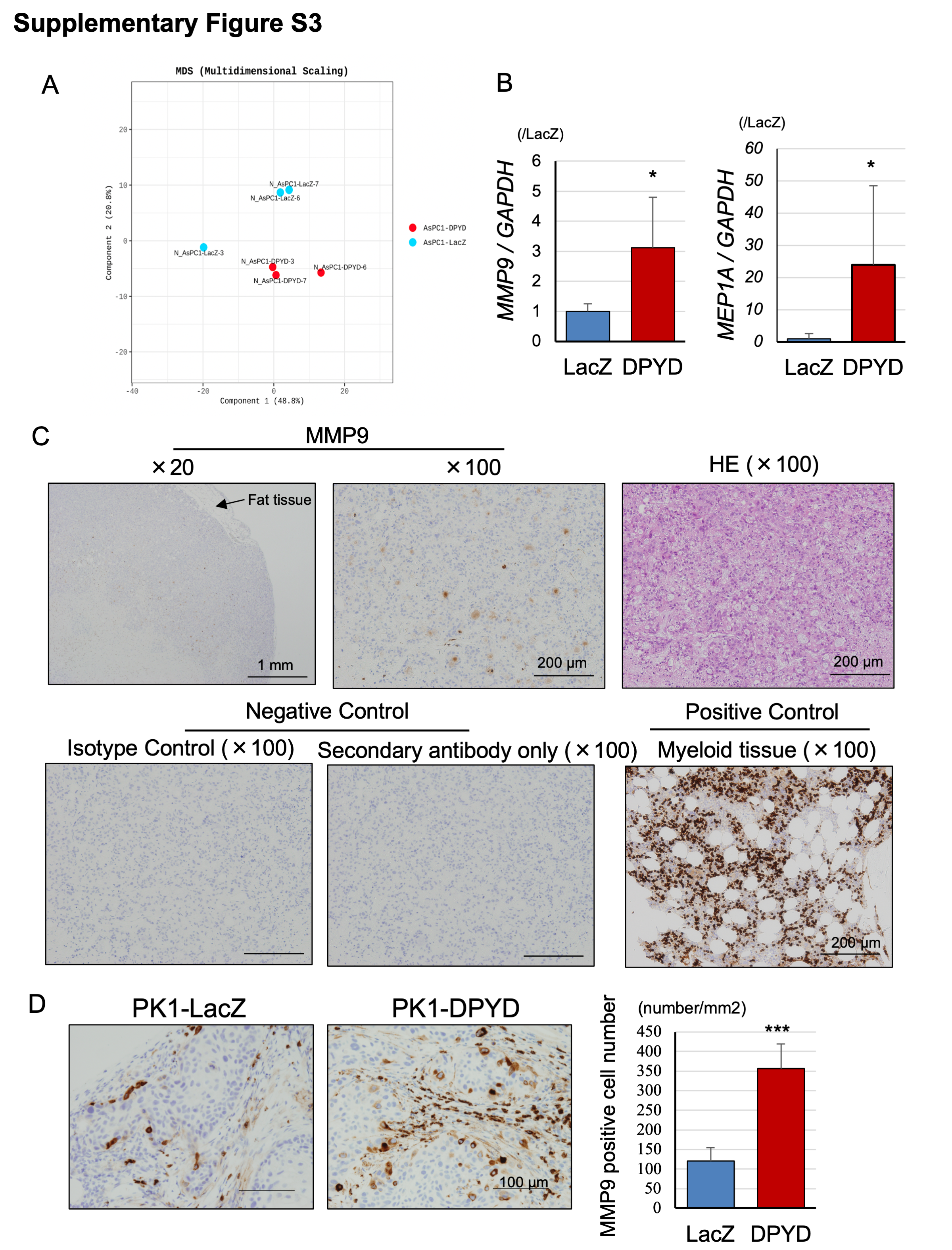

Supplement: Supplementary file 3 — Figure S3. [file CAM4-13-e70124-s007.tiff]

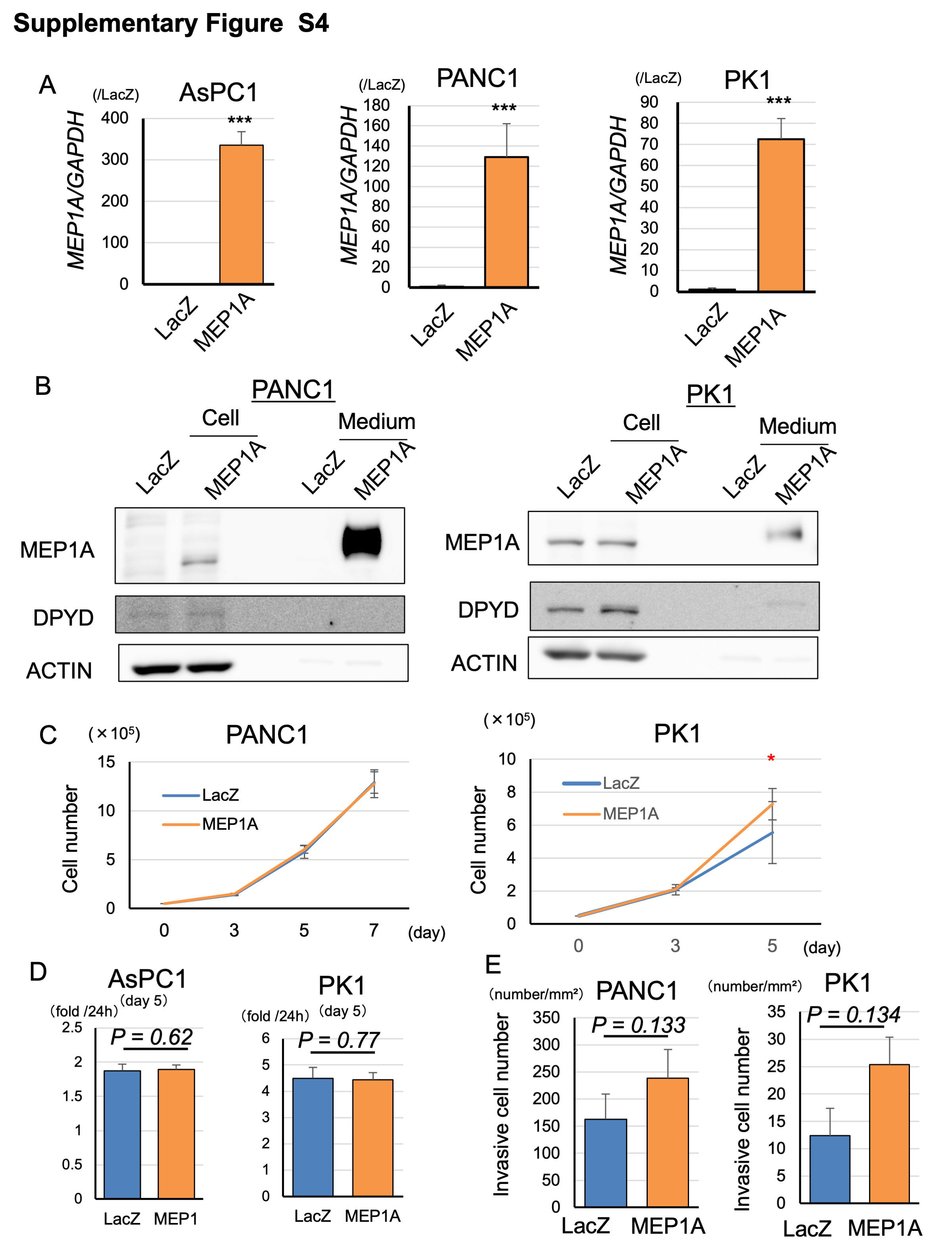

Supplement: Supplementary file 4 — Figure S4. [file CAM4-13-e70124-s011.tiff]

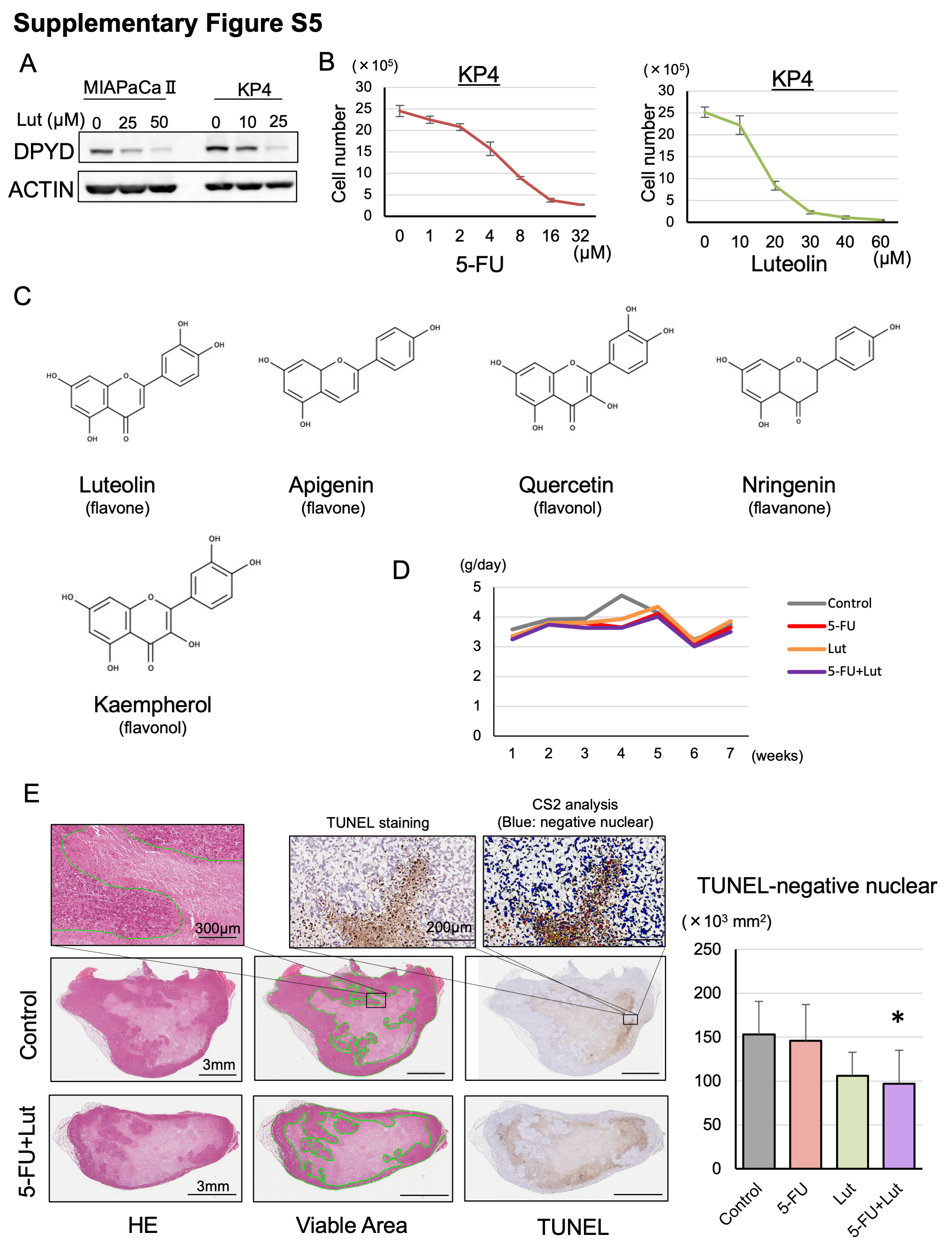

Supplement: Supplementary file 5 — Figure S5. [file CAM4-13-e70124-s006.tiff]

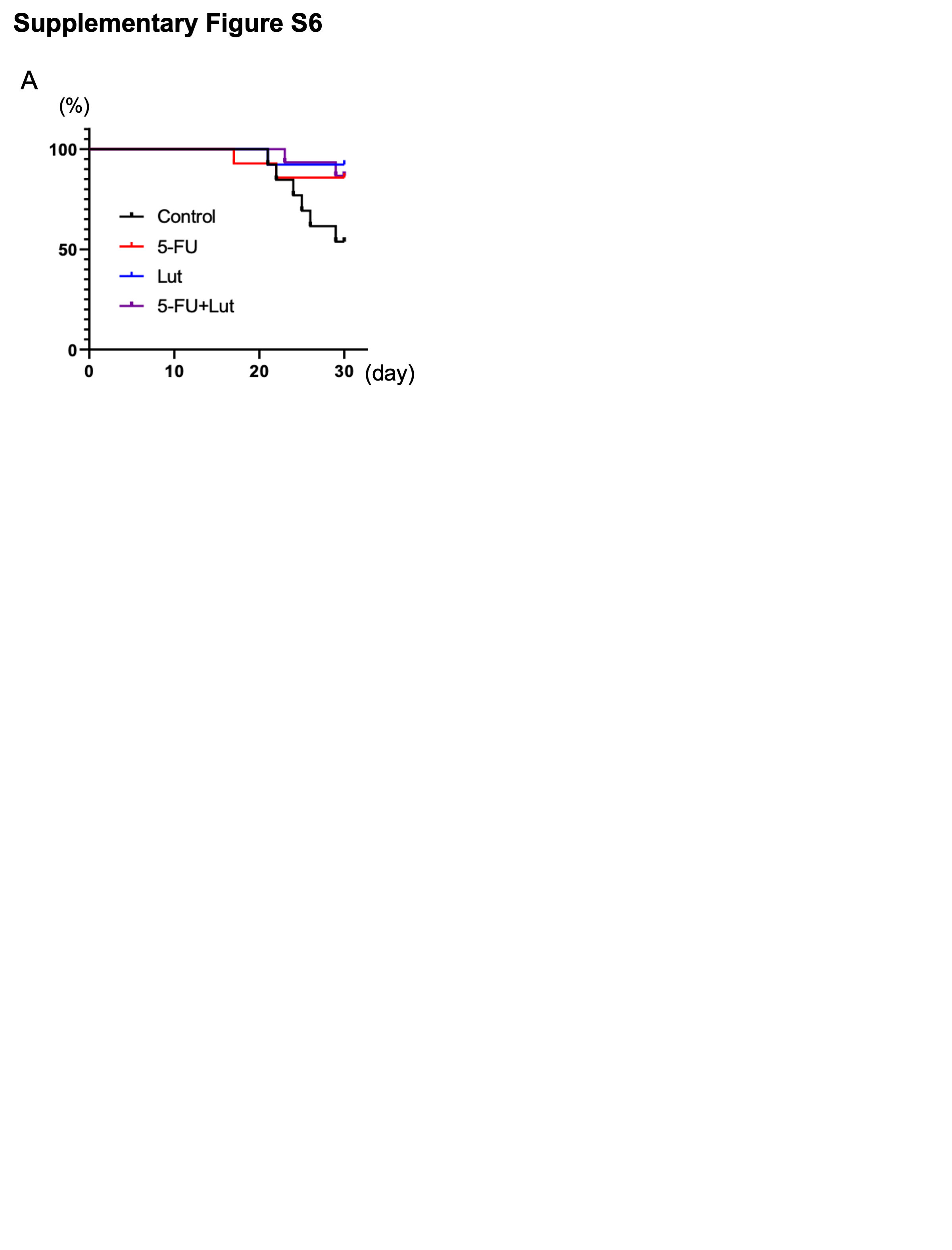

Supplement: Supplementary file 6 — Figure S6. [file CAM4-13-e70124-s002.tiff]

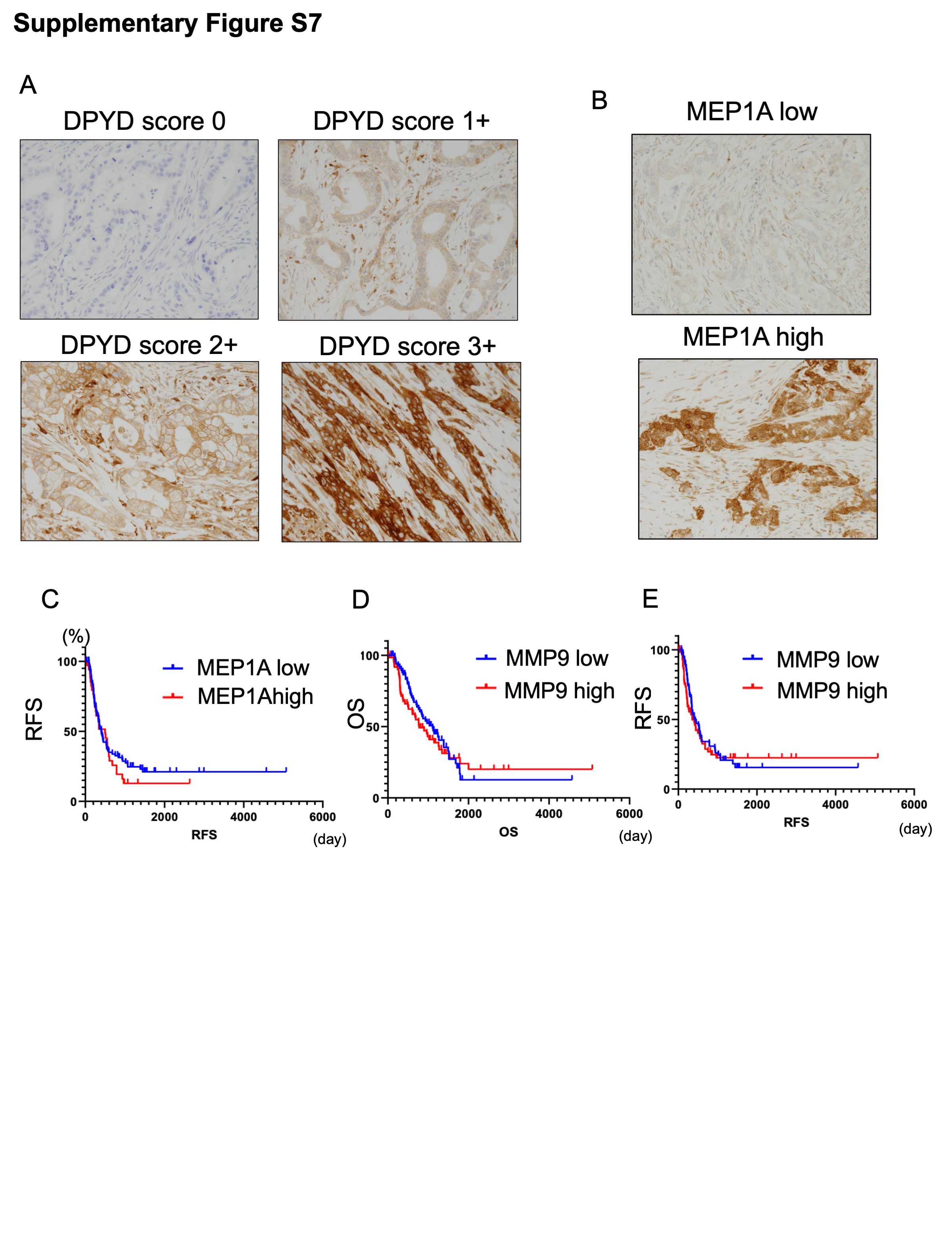

Supplement: Supplementary file 7 — Figure S7. [file CAM4-13-e70124-s012.tiff]
