# Supplementary material for: The role of DPYD and the effects of DPYD suppressor luteolin combined with 5‐FU in pancreatic cancer
Source: Cancer Med. 2024 Aug 19;13(16):e70124. doi: 10.1002/cam4.70124 (PMC11331593; doi:10.1002/cam4.70124)
Supplement: Supplementary file 8 — Table S1. [file CAM4-13-e70124-s001.docx]

Table S1. Primer lists for quantitative RT-PCR

| Species | Gene symbol | Arrangement |
| --- | --- | --- |
| Human | *Xho1-DPYD* | 5'-GGGGGCTCGAGCGCCACCATGGCCCCTGTGCTCAGTAAG-3' |
|  | *EcoRI-DPYD* | 5'-GCGCGGAATTCTTAACACACCGGATTCACAGATAA -3' |
|  | *Nhe1-MEP1A* | 5'-CCCCCGCTAGCCGCCACCATGGCTTGGATTAGATCCACTTGCAT-3' |
|  | *Xho1-MEP1A* | 5'-AAAAACTCGAGTCACTTCCTTGGCCTTTGGGAAAGG-3' |
|  | *DPYD F* | 5'- TGTTCCACTTCGGCCAAGAA -3' |
|  | *R* | 5'- CTCACCAAGAGTCGTGTGCT -3' |
|  | *MEP1A F* | 5'- AGCAGCTGTACCGATTAAGTATCT -3' |
|  | *R* | 5'- TCCTTTAGCATTCAGCCCCAA -3' |
|  | *MMP9 F* | 5'-CGCACGACGTCTTCCAGTA -3' |
|  | *R* | 5’- TGCAGGATGTCATAGGTCACG -3' |
|  | *MEP1B F* | 5'- AGAACTTTCCATCGGGGCAA -3' |
|  | *R* | 5'- AAATTGTGCTCTCTGCCTGACA -3' |
|  | *GAPDH F* | 5'-AGGGCTGCTTTTAACTCTGGT-3' |
|  | *R* | 5'-CCCCACTTGATTTTGGAGGGA-3' |
| Mice | *Dpyd F* | 5'- GACATCAGCGTGGAAATGGC -3' |
|  | *R* | 5'- ACAAGGGGCCAGAAGTTGTC -3' |
|  | *Actin F* | 5'- GGCTCCTAGCACCATGAAGA-3' |
|  | *R* | 5'- AGCTCAGTAACAGTCCGCC-3' |
