# Supplementary material for: The role of DPYD and the effects of DPYD suppressor luteolin combined with 5‐FU in pancreatic cancer
Source: Cancer Med. 2024 Aug 19;13(16):e70124. doi: 10.1002/cam4.70124 (PMC11331593; doi:10.1002/cam4.70124)
Supplement: Supplementary file 9 — Table S2. [file CAM4-13-e70124-s010.docx]

Table S2. Up- and down-regulated genes in AsPC1-DPYD tumors compared to AsPC1-LacZ tumors by RNA-seq analysis (relative gene expression >100)

| Gene symbol | AsPC1-DPYD/LacZ |
| --- | --- |
| *NTN4* | -6.85 |
| *RNA28SN3* | -6.70 |
| *CCDC80* | -6.15 |
| *ZC4H2* | -4.82 |
| *PIWIL4* | -4.58 |
| *MAML3* | -4.21 |
| *ZC3H12B* | -4.17 |
| *STC1* | -4.11 |
| *ADAM28* | -3.97 |
| *AGPAT4* | -3.90 |
| *TGFB2* | -3.72 |
| *FMN1* | -3.53 |
| *SULT1C2* | -3.45 |
| *LRIG1* | -3.42 |
| *FUT4* | -3.30 |
| *MEF2C* | -3.30 |
| *RAB3B* | -3.24 |
| *B4GALT6* | -2.98 |
| *MMP1* | -2.90 |
| *DENND1B* | -2.70 |

Up-regulated genes 　　　　　　　　　　　　Down-regulated genes

| Gene symbol | AsPC1-DPYD/LacZ |
| --- | --- |
| *ACE2* | 10.84 |
| *DSEL* | 7.70 |
| *DPYD* | 5.72 |
| *AKR1B10* | 5.16 |
| *DMKN* | 4.83 |
| *ALDH1A1* | 3.39 |
| *CYP2C9* | 2.99 |
| *CCDC198* | 2.84 |
| *PPL* | 2.82 |
| *NEB* | 2.80 |
| *C3orf85* | 2.77 |
| *DQX1* | 2.74 |
| *HSPA7* | 2.73 |
| *PADI2* | 2.72 |
| *ATP7B* | 2.52 |
| *RAB4B-EGLN2* | 2.49 |
| *MMP9* | 2.48 |
| *IGFL2* | 2.44 |
| *HLA-DMB* | 2.36 |
| *MEP1A* | 2.32 |
