# Supplementary material for: The role of DPYD and the effects of DPYD suppressor luteolin combined with 5‐FU in pancreatic cancer
Source: Cancer Med. 2024 Aug 19;13(16):e70124. doi: 10.1002/cam4.70124 (PMC11331593; doi:10.1002/cam4.70124)
Supplement: Supplementary file 10 — Table S3. [file CAM4-13-e70124-s009.docx]

Table S3. Clinicopathological characteristics of DPYD high or low PDACs

|  | DPYD | | P value |
| --- | --- | --- | --- |
|  | low | high |  |
| n | 91 | 41 |  |
| male/female | 61 / 30 | 27 / 14 |  |
| Age (median) | 69 | 72 |  |
| Differentiation |  |  |  |
| well | 48 (53%) | 7 (17%) |  |
| moderately | 26 (28%) | 14 (34%) |  |
| poorly | 17 (19%) | 20 (49%) | <0.001 |
| Size (mm) |  |  |  |
| 0 – 20 | 23 (25%) | 5 (12%) |  |
| 21 - 40 | 48 (53%) | 26 (63%) |  |
| 41 – 60 | 1. (20%) | 9 (22%) |  |
| >61 | 2 (2%) | 1 (3%) |  |
| N (+) | 55 (60%) | 28 (68%) |  |
| M (+) | 1 (1%) | 2 (5%) |  |
| Resection Margin | 13 (14%) | 10 (24%) |  |
