# Supplementary material for: The role of DPYD and the effects of DPYD suppressor luteolin combined with 5‐FU in pancreatic cancer
Source: Cancer Med. 2024 Aug 19;13(16):e70124. doi: 10.1002/cam4.70124 (PMC11331593; doi:10.1002/cam4.70124)
Supplement: Supplementary file 11 — Table S4. [file CAM4-13-e70124-s004.docx]

Table S4. Clinicopathological characteristics of MMP9 high or low PDACs

|  | MMP9 | | P value |
| --- | --- | --- | --- |
|  | low | high |  |
| n | 70 | 62 |  |
| male/female | 51 / 19 | 37 / 25 |  |
| Age (median) | 69 | 70 |  |
| Differentiation |  |  |  |
| well | 33 (47%) | 24 (39%) |  |
| moderately | 20 (29%) | 19 (31%) |  |
| poorly | 17 (24%) | 19 (31%) |  |
| Size (mm) |  |  |  |
| 0 – 20 | 16 (23%) | 12 (19%) |  |
| 21 - 40 | 38 (54%) | 37 (60%) |  |
| 1. – 60 | 14 (20%) | 11 (18%) |  |
| >61 | 2 (3%) | 2 (3%) |  |
| N (+) | 39 (56%) | 44 (71%) |  |
| M (+) | 1 (1%) | 2 (3%) |  |
| Resection Margin | 14 (20%) | 9 (15%) |  |
| DPYD score 0 | 7 (10%) | 3 (5%) |  |
| 1+ | 31 (44%) | 23 (37%) |  |
| 2+ | 17 (24%) | 12 (19%) |  |
| 3+ | 15 (21%) | 24 (39%) | <0.01 |
