# Supplementary material for: The role of DPYD and the effects of DPYD suppressor luteolin combined with 5‐FU in pancreatic cancer
Source: Cancer Med. 2024 Aug 19;13(16):e70124. doi: 10.1002/cam4.70124 (PMC11331593; doi:10.1002/cam4.70124)
Supplement: Supplementary file 13 — Supplementary Materials and Methods. [file CAM4-13-e70124-s003.docx]

**Supplementary Materials and Methods.**

*Cell proliferation and 5-FU resistance assay by cell counts*

AsPC1-LacZ, DPYD, MEP1A, and PK1-LacZ, DPYD, MEP1A cells were seeded at a density of 1.0×10^5^ cells in 6-well plates, and cell counts were measured using a OneCell counter (OneCell, Hiroshima, Japan) at the indicated time points (n=4). For the assessment of 5-FU resistance, ASPC1-LacZ, DPYD, and PK1-LacZ, DPYD cells were seeded at a density of 1.0×10^5^ cells in 6-well plates and treated with 5-FU (AsPC1: 0-4μM, PK1:0-16μM) the following day. After an additional 72 hours, the cells were counted using a OneCell counter (n=3−4).

*Cell proliferation and 5-FU resistance assay by WST-1*

To investigate the proliferative ability of AsPC1-LacZ, DPYD, MEP1A, and PK1-LacZ, DPYD, MEP1A, we seeded each cell line at 1.0×10^4^ (AsPC1-) and 5.0×10^3^ (PK1-) cells/100 μl RPMI medium without phenol red (Thermo Fisher Scientific) in 96-well plates (n=8). On Day 1 (24 hours later), 10 μl of WST-1 reagent (Roche Ltd., Basel, Switzerland) were added to each well, and the absorbance was measured at 460 nm and 650 nm after 3−4 hours. The absorbance at 24 hours (460−650 nm) was used as the baseline, and the absorbance on Day 5 was measured and compared to the 24-hour absorbance. Next, to investigate the resistance to 5-FU treatment, cells were seeded using the same protocol. After 24 hours, 100 μl of medium containing 5-FU was added to achieve final concentrations of 0−8 μM for AsPC1 and 0−16 μM for PK1. Subsequently, 10 μl of the WST-1 reagent were added after 4 or 6 days. The absorbance was measured using the same protocol as the one used to investigate cell proliferation to assess the growth inhibitory effect compared to untreated cells (0 μM).

*Invasion Assay*

The invasion assay was performed using the Corning BioCoat Matrigel invasion chamber (8μm-pore, 24 well, Corning, NY, USA). AsPC1-LacZ and DPYD cells were seeded at a density of 5.0×10^4^ cells in the upper chamber without FBS-containing medium and stimulated for 72 hours using 5% FBS in the lower chamber. As for AsPC1-LacZ and MEP1A cells, AsPC1 cells were seeded at a density of 5.0×10^4^ cells. Then, the upper chamber was filled with culture medium (FBS-) from AsPC1-LacZ and MEP1A cells after 48 hours of culture, and the lower chamber was stimulated for 72 hours using 5% FBS. Subsequently, the membrane was stained with Giemsa, and the number of invading cells per unit area was counted.

*Tumor-initiating cell-frequency*

To investigate the stemness of AsPC1-LacZ and DPYD cells, 10^3^, 10^4^, 10^5^, and 10^6^ cells were subcutaneously transplanted into KSN nu/nu mice (n=7−8 per group). The mice were observed for 8 weeks, and the number of individuals with tumor formation was counted. Using the number of transplanted cells and the number of individuals with tumor formation, the tumor-initiating cell frequency was estimated using Extreme Limiting Dilution Analysis which was explained previously in *Journal of Immunological Methods* in 2009 ^13^.

*RNA-seq analysis of xenograft tumors*

Total RNA was extracted from xenograft tumors of AsPC1-LacZ (n=3) and AsPC1-DPYD (n=3) using the ISOGEN (Nippon Gene Co., LTD, Toyama, Japan).　RNA integrity was assessed using RIN. RNA-seq libraries were prepared using the TruSeq Stranded Total RNA LT Sample Prep Kit, and libraries were sequenced on the illumina platform with 100bp paired-end reads. Raw sequencing reads were quality-checked using FastQC. Adapters and low-quality bases were trimmed using Trimmomatic 0.38. Reads were aligned to the Graft (GRCh38) and Host (mm10) reference, respectively, using BAMCMP. Graft-only reads were used to extract gene expression profiles for each sample. Known genes and transcripts are assembled with StringTie based on reference genome model. The abundance of a gene was calculated in the read count and normalized values expressed as Fragments Per Kilobase of transcript per Million mapped reads and Transcripts Per Kilobase Million for sample. From a total of 46,427 genes, 17,745 genes that were detectable in any of the samples were extracted, and normalization was performed using the Relative Log Expression method within the DESeq2 library. Analysis was conducted using the processed data (Supplementary data 1). Using each sample’s normalized value, the similarity between samples was graphically shown in a 2D plot (Multidimensional Scaling Analysis) to show the variability of the total data (Figure S3A). Statistical analysis was performed using fold change, nbinomWaldTest using DESeq2 per comparison pair. The significant results were selected on conditions of |fc| >=2 and nbinomWaldTest raw p-value < 0.05. For significant lists (227 genes), hierarchical clustering analysis was performed to group the similar samples and genes. These results were graphically depicted using heatmaps and dendograms (Figure 2A). Gene-set enrichment analysis was performed based on gene ontology (https://biit.cs.ut.ee/gprofiler) (Figure 2B).

*Western blotting*

Cells or tissue were homogenized with T-PER Tissue Protein Extraction Reagent (Thermo Fischer Scientific Inc.) in the presence of a protease inhibitor cocktail (Roche Diagnostic, Mannheim, Germany) on ice. Protein concentrations were determined by the Bradford method using protein assay kits (Bio-Rad laboratories, Hercules, CA). Samples of 30 μg were mixed with SDS sample buffer, heated for 10 min at 100°C and then subjected to SDS-PAGE. The proteins separated on the 8-12% acrylamide gels were transferred onto Hybond-ECL membranes (GE Healthcare UK Ltd., Buckinghamshire, UK). The antibodies used were DPYD (Abcam, 1:1000) and MEP1A (R&D, 1:1000). Equal protein loading was ascertained by Western blotting with β-actin antibody (Sigma-Aldrich corp., St. Louis, MO).

*RNA extraction and quantitative reverse transcription PCR*

Total RNA was isolated from pancreatic gastric lobe tissues by phenol-chloroform extraction (Isogen, Nippon Gene Co. Ltd., Tokyo, Japan). One microgram of RNA was converted to cDNA with avian myoblastosis virus reverse transcriptase (Takara, Otsu, Japan) in 12μl reaction mixture. Aliquots of 1 μl of cDNA samples were subjected to quantitative PCR in a total volume of 25 μl using SYBR Premix ExTaq II (Takara) in a light cycler apparatus (Roche DiagnosticBasel, Switzerland). The primers used are listed in Supplementary Table 1.

Combination Index in vitro

We calculated the combination index (CI) by using the Chou-Talalay Method^14^. To calculate the Combination Index (CI), KP4 cells were seeded at a density of 1.0×104 cells and treated with varying concentrations of Lut (0, 10, 20, 30, 60μM) and 5-FU (0, 1, 4, 6, 8μM) for 48 hours. Subsequently, viable cells were counted, and a graph representing the therapeutic effects was created. Simultaneously, combination treatment of 5-FU and Lut (at concentrations of 5-FU/Lut: 1/10, 1/15, 2/10, 2/15μM) was performed, and the CI was calculated using the formula: CI = [Combined 5-FU concentration]/[Individual 5-FU concentration required for treatment] + [Combined Lut concentration]/[Individual Lut concentration required for treatment]. A CI value greater than 1.1 indicated antagonism, 0.9 < CI < 1.1 indicated additivity, and CI < 0.9 indicated synergy.

*Luteolin analogue*

We prepared apigenin (TCI), quercetin (TCI), kaempferol (TCI), and naringenin (TCI) as Lut analogues from flavonoids. All compounds were treated with KP4 cells at a concentration of 25 μM for 48 hours, and the cells were collected to assess the expression of DPYD by Western blotting.
